# Supplementary material for: HIRA defines early replication initiation zones independently of their genome compartment
Source: Nat Commun. 2025 Nov 6;16:9715. doi: 10.1038/s41467-025-65130-2 (PMC12592364; doi:10.1038/s41467-025-65130-2)
Supplement: Supplementary file 1 — Supplementary Information [file 41467_2025_65130_MOESM1_ESM.pdf]

# HIRA defines early replication initiation zones independently of their genome compartment

T. Karagyozova[1,6], A. Gatto[1], A. Forest[1], J.-P. Quivy[1], R. Nunez-Vazquez[1], M. Marti-Renom[2,3,4], L. Mirny[5] & G. Almouzni[1]

[1]Institut Curie, PSL Research University, Sorbonne Université, CNRS UMR3664, Laboratoire Dynamique du Noyau, Equipe Labellisée Ligue contre le Cancer, 75005 Paris, France

[2] Centre Nacional d'Anàlisi Genòmica (CNAG), Baldiri i Reixac 4, 08028 Barcelona, Spain

[3] Centre for Genomic Regulation (CRG), Barcelona Institute of Science and Technology (BIST), Dr. Aiguader 88, 08003 Barcelona, Spain

[4] ICREA, Pg. Lluís Companys 23, 08010 Barcelona, Spain

[5] Institute for Medical Engineering and Science, and Department of Physics, Massachusetts Institute of Technology, Cambridge, MA 02139, USA

[6]Present address: Institute of Cell Biology, School of Biological Sciences, University of Edinburgh, Roger Land Building, Alexander Crum Brown Road, Edinburgh, EH9 3FF, UK

Corresponding author: genevieve.almouzni@curie.fr

## Supplementary Information

Supplementary Information are provided as a single pdf file and contain:

- Supplementary Figures 1-7
- Supplementary Figure Legends 1-7
- Supplementary Tables 1-3

# Supplementary Figure 1.

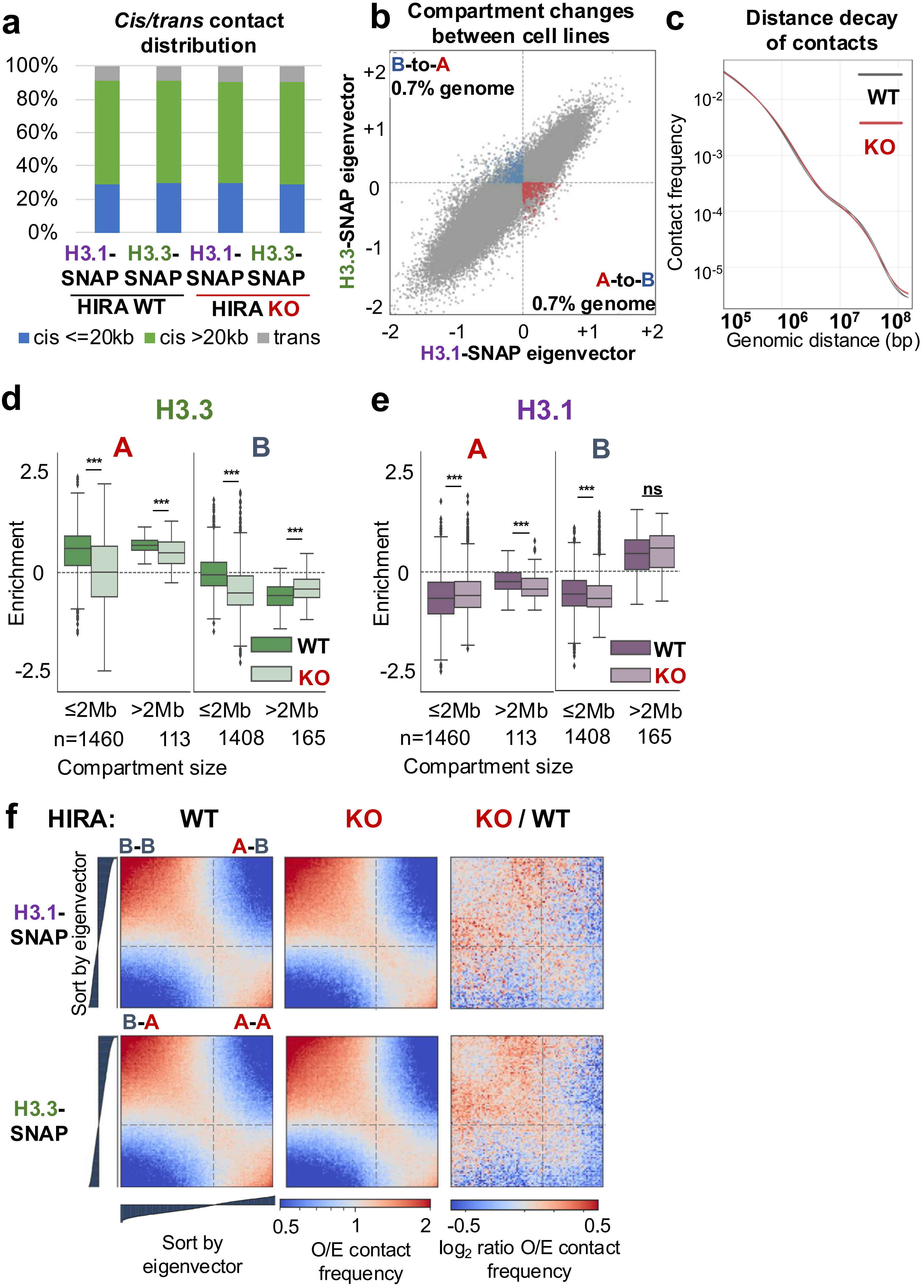

**Supplementary Figure 2.**

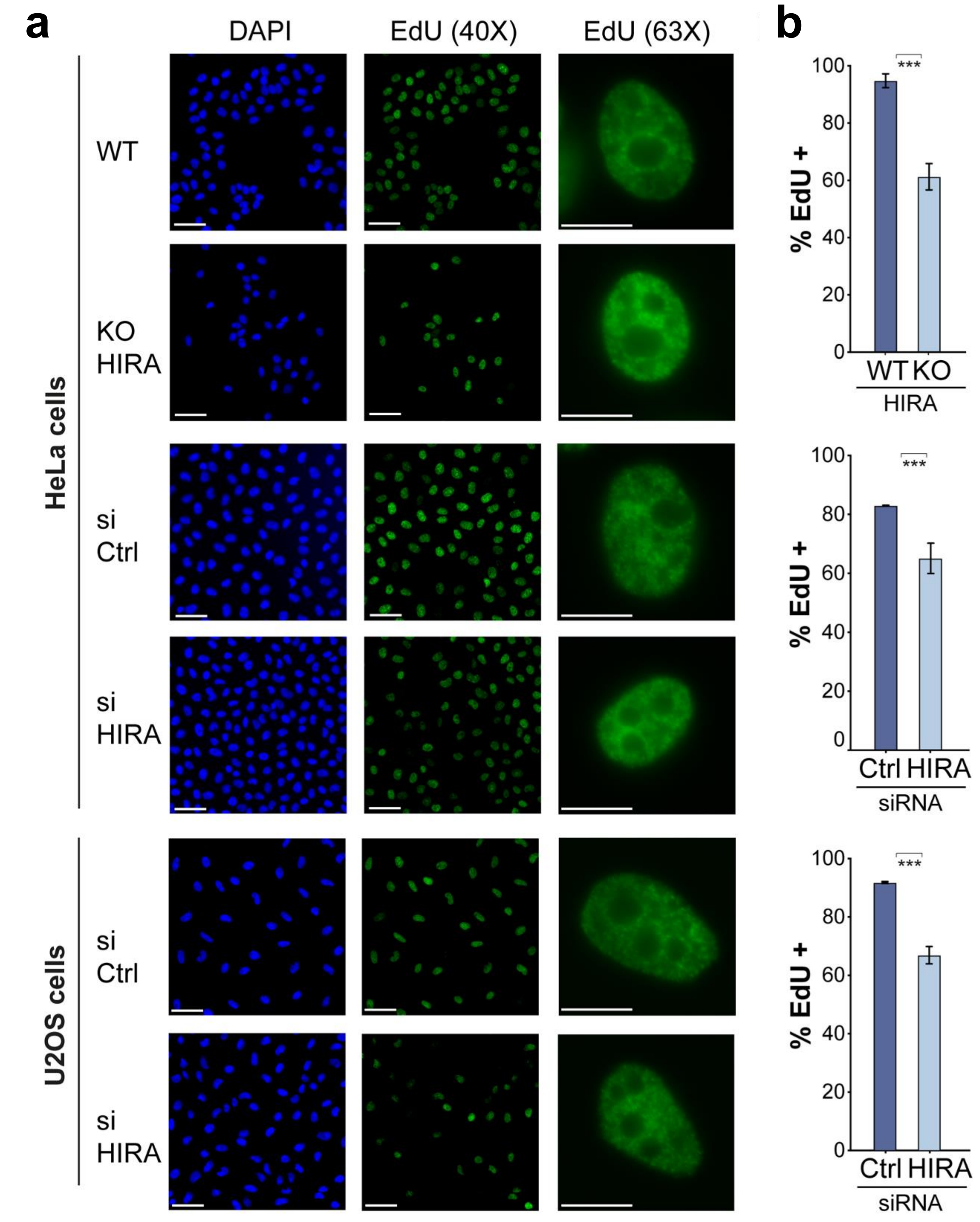

**Supplementary Figure 3.**

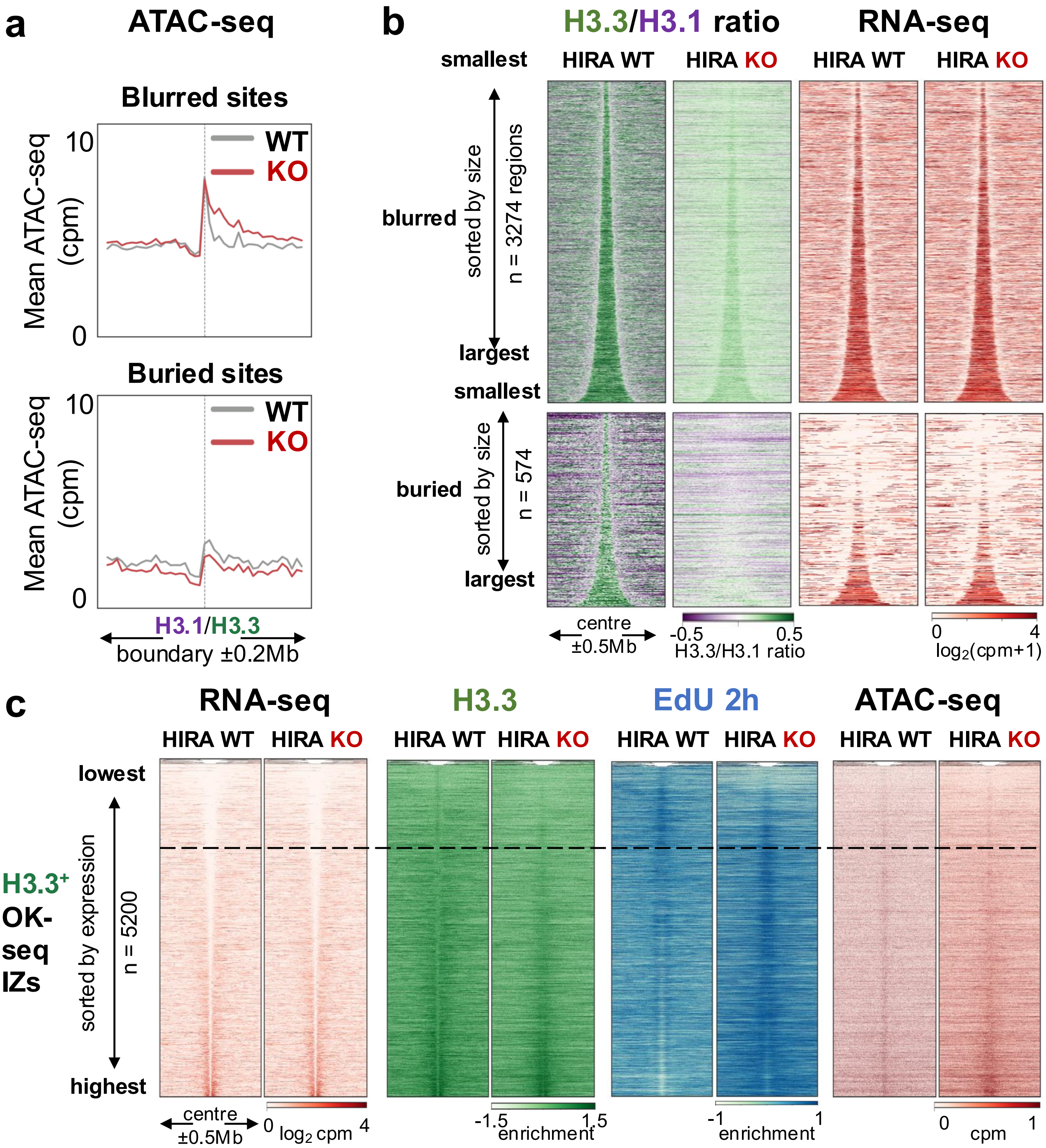

# Supplementary Figure 4.

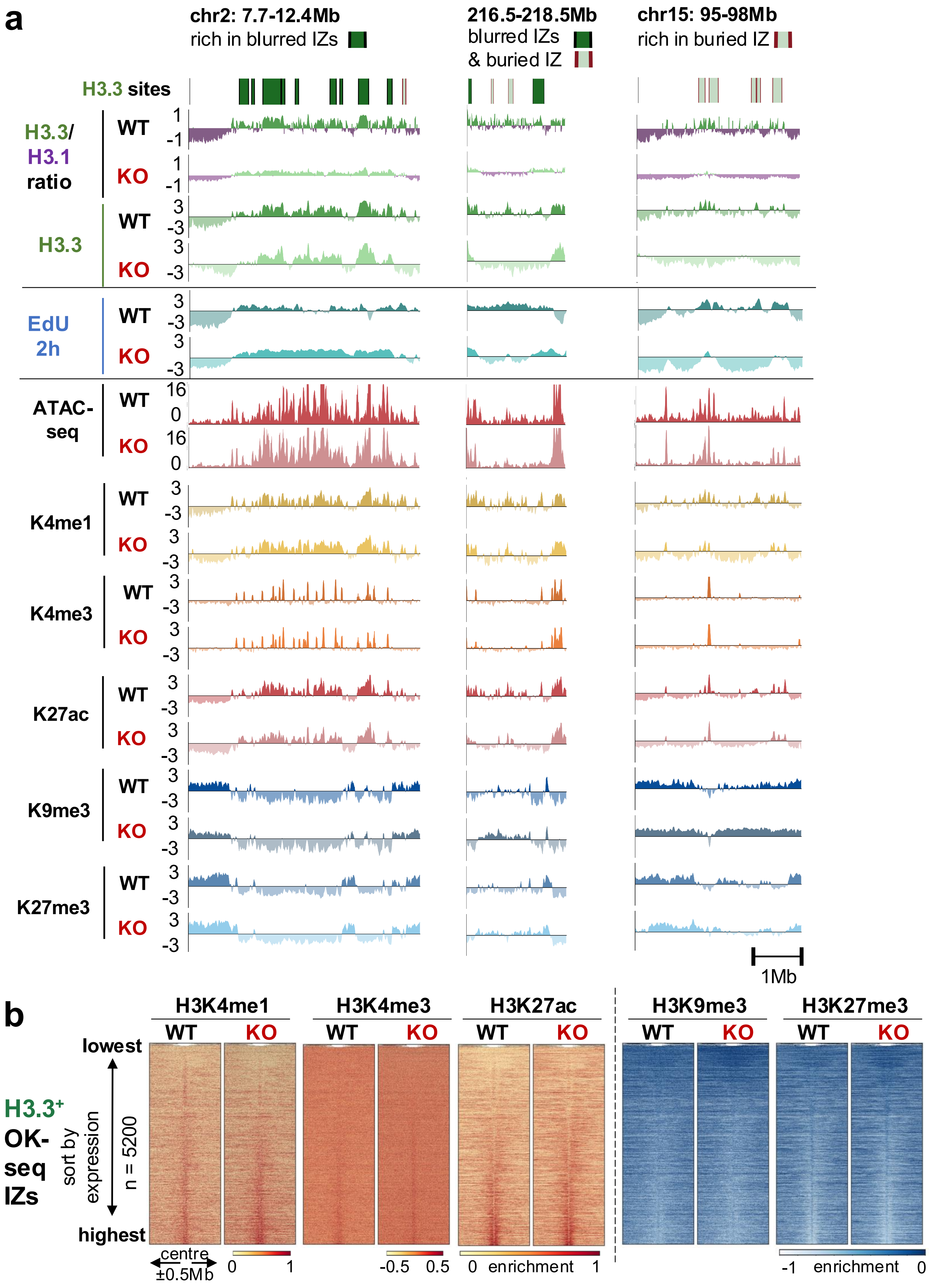

## Supplementary Figure 5.

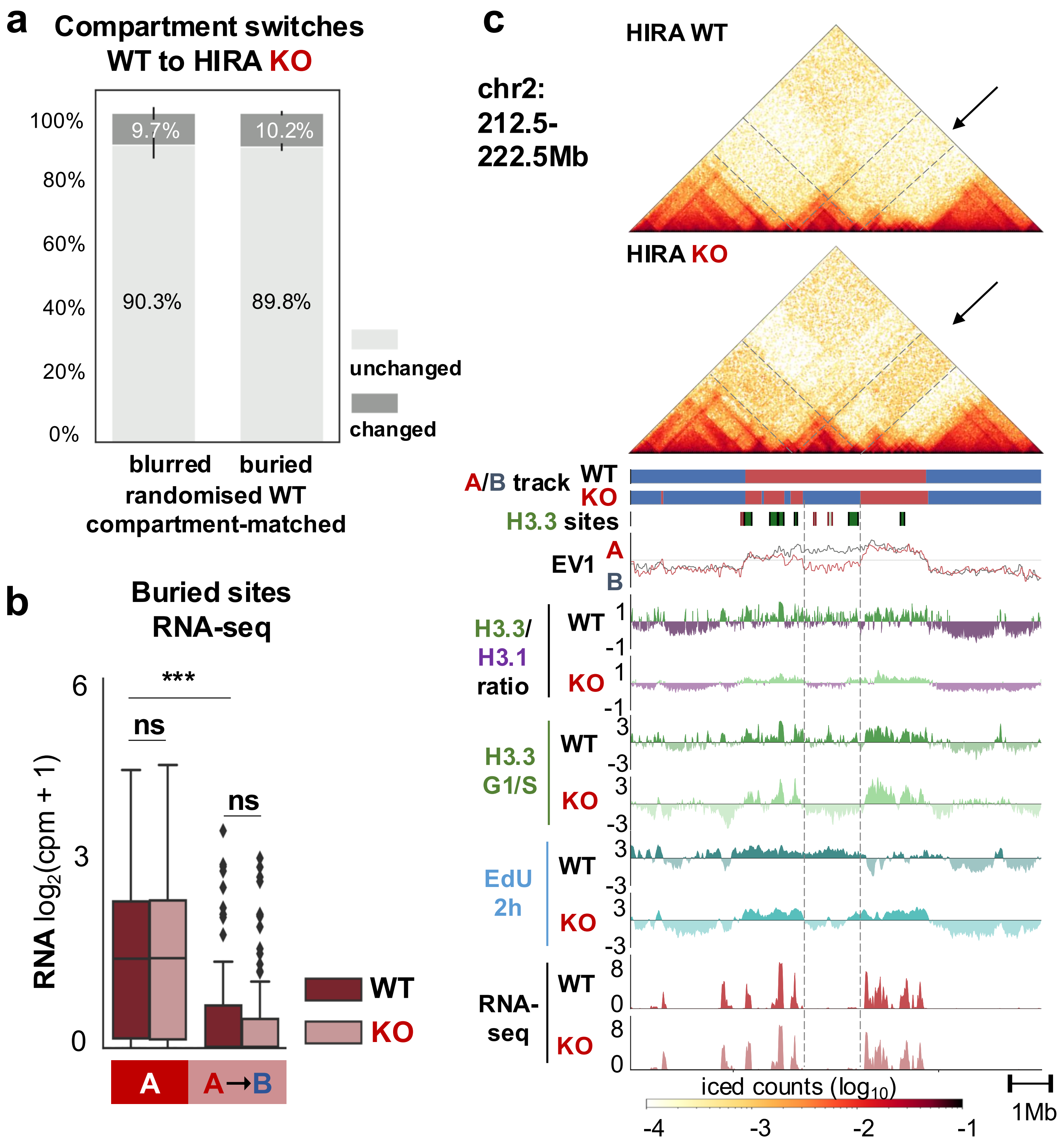

# Supplementary Figure 6.

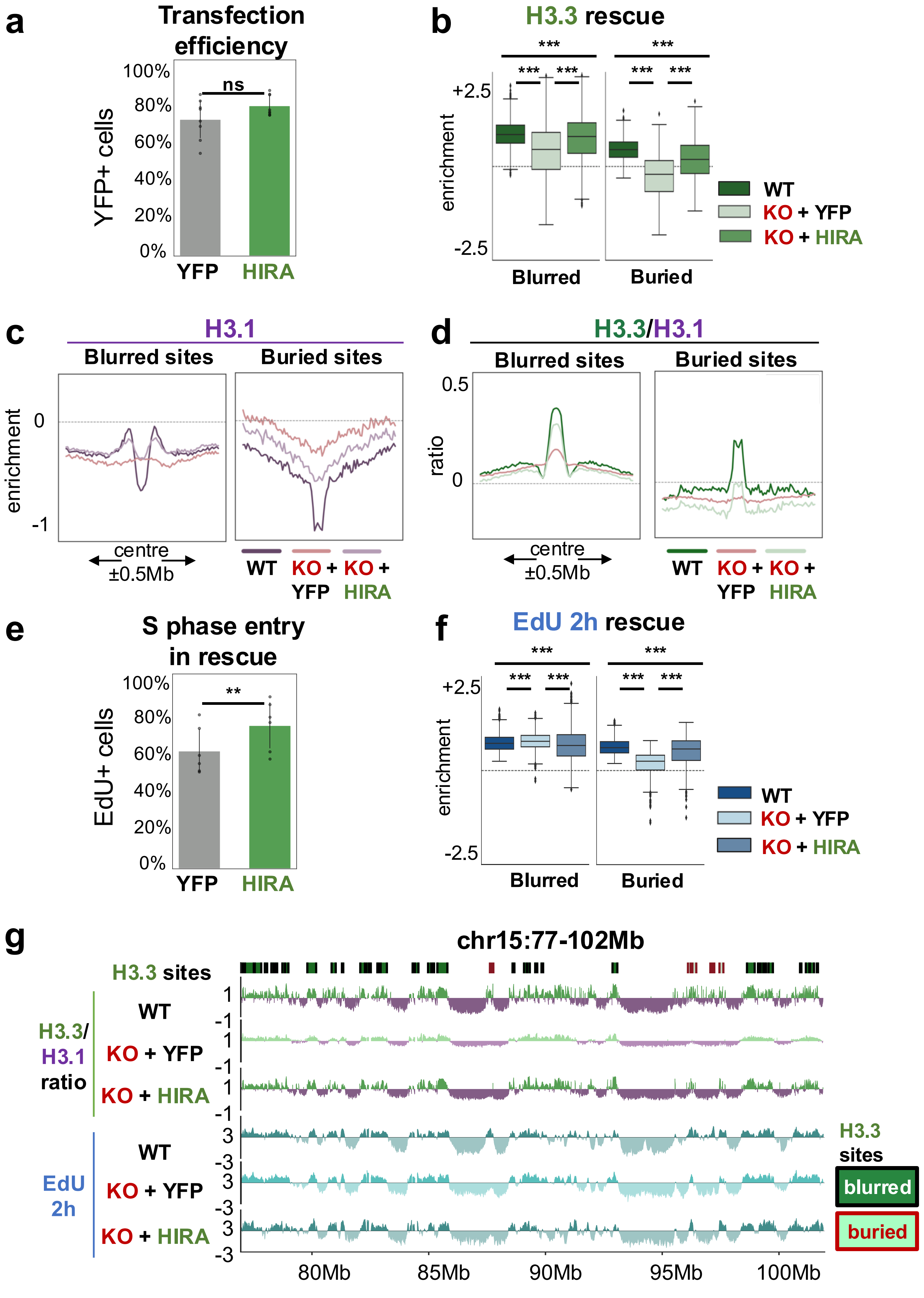

# Supplementary Figure 7.

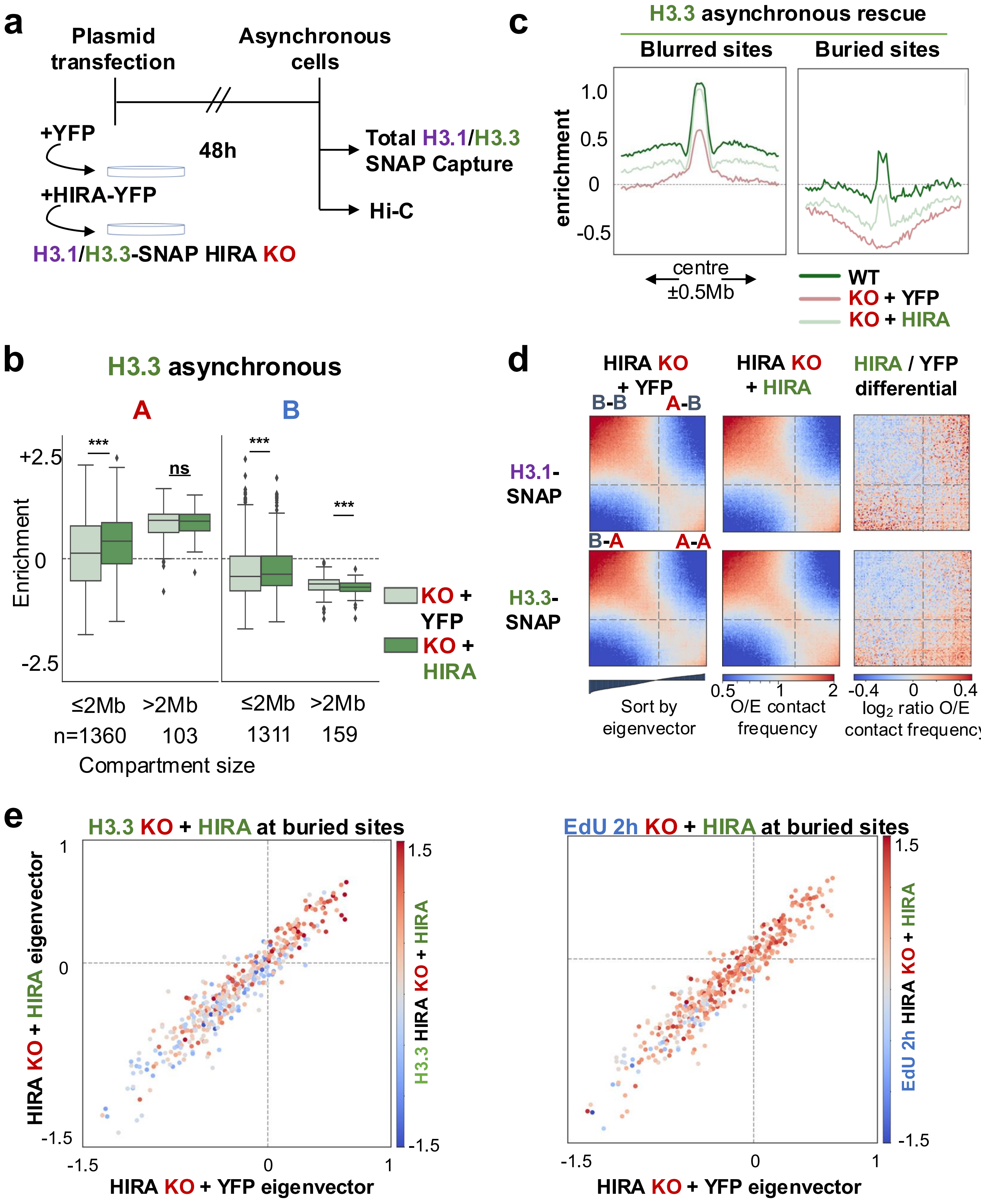

## Supplementary Figure Legends

**Supplementary Figure 1. Absence of HIRA leads to re-distribution of H3.3 from A to large B compartments**

**A.** Proportion of short-range (<=20kb, blue), long-range (>20kb, green) *cis* and *trans* (grey) contacts from Hi-C maps of HIRA WT or KO H3.1- and H3.3-SNAP HeLa cells. Source data are provided as a Source data file.

**B.** EV1 ($1^{st}$ eigenvector, indicating compartment) of 50kb-binned Hi-C matrices from H3.1- vs H3.3-SNAP HIRA WT cells. Bins which change from A-to-B (lower right quadrant) or B-to-A (upper left quadrant) in the same direction in both conditions are coloured red and blue, respectively.

**C.** Decay of contact frequency with genomic distance (P(s) curves) from HIRA WT (grey) and KO (red) maps, binned at 10kb after masking of blacklisted regions and ICE normalization.

**D.** H3.3 and **E.** H3.1 enrichment from WT (darker) and HIRA KO (lighter) cells quantified in A/B compartments per indicated domain sizes. Two-tailed Mann-Whitney U test adjusted for multiple testing by FDR (5% cut-off) was used to determine significance of differences between WT and KO. The boxplot centre is the median, the bounds of the box are the first and third quartiles and the whiskers extend to 1.5x IQR. Significance (2-tailed Mann-Whitney U test): ns, p>0.05; *, p<=0.05; **, p<=0.01; ***, p<=0.001. P-values are for WT vs HIRA KO H3.3: A<=2Mb (p=1.63e$^{-73}$), A>2Mb (p=3.11e$^{-06}$), B<=2Mb (p=2.47e$^{-94}$), B>2Mb (p=2.29e$^{-08}$) and H3.1: A<=2Mb (p=1.64e$^{-07}$), A>2Mb (p=0.0006), B<=2Mb (p=0.0004), B>2Mb (p=0.16). Source data are provided as a Source data file.

**F.** Saddle plots from HIRA WT and KO (O/E contacts) and differential ($log_2$ ratio of O/E contacts) of HIRA KO/WT at 50kb resolution based on EV1 percentiles from H3.1- and H3.3-SNAP cells.

H3.3 and H3.1 enrichment is shown as z-score of $log_2$ IP/input at 10kb bins.

**Supplementary Figure 2. HIRA depletion impairs S-phase entry in HeLa and U2OS cells.**

**A.** Top: Representative images of HeLa cells showing EdU incorporation (green) and DNA staining (DAPI, blue) under different HIRA depletion strategies: genetic ablation (HIRA KO) and siRNA silencing (siHIRA), compared to their respective controls (WT or siCtrl). Bottom: Representative images of U2OS cells transfected with control or HIRA-targeting siRNAs and stained for EdU and DAPI. Images were acquired following release from a double thymidine block and EdU pulse labeling at early S-phase time points (2 h post-release for HeLa cells and 5 h post-release for U2OS cells). For each condition, high-magnification insets of single nuclei are shown on the right to highlight the consistent early S-phase replication pattern observed across cells. Scale bars, 50 µm (lower magnification) and 10 µm (higher magnification).

**B.** Quantification of EdU-positive cells expressed as a percentage of total cells after release from double thymidine block from HIRA WT (dark blue) or depleted (light blue) cells. Statistical comparisons were performed using unpaired two-sided Welch's test (** $p < 0.005$, *** $p < 0.001$). Data represent individual cells (n=1000 cells per condition), derived from two biological replicates per group. Data are presented as mean ± standard deviation (SD). HeLa cells (WT vs HIRA KO): $p=9.74e^{-5}$. HeLa cells (siCtrl vs siHIRA): p=0.0032. U2OS cells (siCtrl vs siHIRA): p=0.00041. Source data are provided as a Source data file.

**Supplementary Figure 3. HIRA defines early replication initiation zones independently of its importance for chromatin accessibility**

**A.** Mean ATAC-seq signal at 10kb bins from WT (grey) and HIRA KO (red) cells centered at the H3.1/H3.3 boundaries of blurred (n = 3274) and buried (n = 574) sites ±0.2Mb.

**B.** H3.3/H3.1 ratio and RNA-seq at 10kb bins from G1/S-arrested WT and HIRA KO cells at blurred and buried sites, sorted by size and centered at their middle ±0.5Mb.

**C.** RNA-seq, enrichment of H3.3, EdU at 2h in S and ATAC-seq signal at 1kb bins from WT and HIRA KO cells at $H3.3^{+}$ OK-seq IZs (n = 5596, as classified in Gatto et al., 2022[19]), sorted by RNA-seq signal and centered at their middle ±0.5Mb. The dashed line represents bottom quartile of expression.

H3.1, H3.3 and EdU 2h enrichment relative to input was calculated at 1kb bins as z-score of $\log_2$ IP/input. RNA-seq is plotted as $\log_2$(cpm+1) and ATAC-seq as cpm.

**Supplementary Figure 4. Impaired early IZ firing in the absence of HIRA is not associated with local H3 PTM redistribution**

**A.** H3.3/H3.1 ratio, H3.3 enrichment, early replication (EdU 2h), ATAC-seq signal (cpm) and enrichment of active (H3K4me1, H3K4me3, H3K27ac) and repressive (H3K9me3, H3K27me3) histone PTMs at 10kb bins from WT and HIRA KO cells at representative chromosomal regions containing blurred and buried sites (denoted above the tracks in dark and light green, respectively).

**B.** Active (H3K4me1, H3K4me3, H3K27ac) and repressive (H3K9me3, H3K27me3) histone PTM enrichment at 1kb bins from WT and HIRA KO cells at $H3.3^{+}$ OK-seq IZs (n = 5596, as classified in Gatto et al., 2022[19]), sorted by RNA-seq signal and centered at their middle ±0.5Mb.

Enrichment relative to input was calculated as z-score of $\log_2$ IP/input. In the representative tracks, signals are shown at 10kb bins smoothed over 3 non-zero bins.

**Supplementary Figure 5. In the absence of HIRA, only non-transcribed early initiation zones switch from A to B compartment**

**A.** Proportion of a size- and WT compartment assignment-matched set of blurred or buried sites which remain in the same compartment (unchanged, light grey) or undergo a switch (changed, dark grey) from WT to HIRA KO cells.

**B.** RNA-seq at buried sites which remain in compartment A (n = 92) or switch form A-to-B (n = 92) form WT (dark red) to HIRA KO (light red) cells. The boxplot centre is the median, the bounds of the box are the first and third quartiles and the whiskers extend to 1.5x IQR. Two-tailed Mann-Whitney U test corrected for multiple testing by FDR (5% cut-off) was used to determine significance of differences between WT and KO. Significance was noted as: * ($p<=0.05$), ** ($p<=0.01$), *** ($p<=0.001$) for all comparisons. P-values WT vs HIRA KO at A-to-A sites (p=0.84), A-to-B sites (p=0.84), A-to-A vs A-to-B sites WT ($p=7.75e^{-9}$) and HIRA KO ($p=3.31e^{-8}$). Source data are provided as a Source data file.

**C.** Hi-C maps from WT and HIRA KO cells at a representative region switching compartment (chr2: 212.5-222.5Mb). Compartment track and blurred and buried site locations (dark and light green boxes, respectively) are noted below. EV1 signal (WT, grey, and HIRA KO, red), H3.3/H3.1 ratio, H3.3 enrichment, EdU at 2h in S and RNA-seq from WT and HIRA KO cells are shown at 10kb bins smoothed over 3 non-zero bins. Grey vertical lines and arrows denote a cluster of buried sites which are not transcribed and switch from compartment A to B concomitantly with losing EdU incorporation at 2h in S and H3.3 enrichment in HIRA KO.

H3.3, H3 PTM and EdU enrichment are calculated as z-score of $log_2$ IP/input ratio of cpm and RNA-seq is shown as $log_2$(cpm+1), all at 10kb resolution.

**Supplementary Figure 6. HIRA rescue reestablishes H3 variant pattern and early firing at blurred and buried sites**

**A.** Proportion of YFP positive cells following transfection of HIRA KO cells with YFP (control, grey) or HIRA-YFP (green). Mean, standard deviation and values of 8 independent experiments are shown. Source data are provided as a Source data file.

**B.** H3.3 enrichment from WT (as reference, dark green) and HIRA KO cells rescued with YFP (control, light green) or HIRA (green) at blurred (n = 3274) and buried (n = 574) sites. P-values for blurred sites are $p=6.89e^{-221}$ (WT vs KO+YFP), $p=9.42e^{-100}$ (KO+YFP vs KO+HIRA), $p= 1.48e^{-18}$ (WT vs KO+HIRA) and for buried sites are $p=2.75e^{-103}$ (WT vs KO+YFP), $p=1.04e^{-33}$ (KO+YFP vs KO+HIRA), $p=1.03e^{-21}$ (WT vs KO+HIRA). Source data are provided as a Source data file.

**C.** H3.1 enrichment from WT (as reference, purple), and HIRA KO cells rescued with YFP (control, red) and HIRA (light purple) at blurred and buried sites between 60-160kb in length, centred in their middle ±0.5Mb.

**D.** H3.3/H3.1 ratio plotted as described for H3.1 enrichment above.

**E.** Proportion of cells in early S phase 2h post-release from G1/S arrest from HIRA KO cells transfected with YFP (control, grey) or HIRA-YFP (green). Mean, standard deviation and values of 6 independent experiments are shown. Source data are provided as a Source data file.

**F.** EdU at 2h in S (right) enrichment from WT (as reference, dark blue) and HIRA KO cells rescued with YFP (control, light blue) or HIRA (blue) at blurred and buried sites. P-values for blurred sites are p= 2.69e$^{-29}$ (WT vs KO+YFP), p=4.15e$^{-55}$ (KO+YFP vs KO+HIRA), p=9.91e$^{-18}$ (WT vs KO+HIRA) and for buried sites are p=7.80e$^{-106}$ (WT vs KO+YFP), p=3.73e$^{-43}$ (KO+YFP vs KO+HIRA), p=1.86e$^{-5}$ (WT vs KO+HIRA). Source data are provided as a Source data file.

**G.** H3.3/H3.1 ratio and EdU at 2h in S from WT (as reference) and HIRA KO cells rescued with YFP (control) and HIRA along a representative region (chr15:77-102Mb) containing blurred and buried sites (denoted in dark and light green on top of the tracks, respectively).

H3.3 and EdU enrichment were calculated at 10kb bins as z-score of $log_2$ IP/input. Two-tailed Mann-Whitney U test corrected for multiple testing by FDR (5% cut-off) was used to determine significance of differences between WT, HIRA KO + YFP (control) and HIRA KO + HIRA rescue. Paired t-test was used to determine significance of differences between proportion of YFP+ or EdU+ cells following HIRA or YFP transfection. Significance was noted as: * (p<=0.05), ** (p<=0.01), *** (p<=0.001) for all comparisons. P-values for transfection efficiency (S6a, p=0.15) and release in S efficiency (S6e, p=0.003). In the boxplots, the centre is the median, the bounds of the box are the first and third quartiles and the whiskers extend to 1.5x IQR.

**Supplementary Figure 7. HIRA rescue recovers H3.3 enrichment and early firing at buried sites without compartment reversal**

**A.** Scheme of experimental strategy to perform HIRA rescue in asynchronous cells to assay total H3.1/H3.3-SNAP and 3D genome organisation. Asynchronous cells constitutively expressing H3.1- or H3.3-SNAP were transfected with YFP (control) or HIRA-YFP plasmid for 48h. Total H3.1- and H3.3-SNAP were assayed by SNAP-Capture ChIP-seq of native MNase-digested chromatin, with matching inputs collected. Compartment organisation was assayed by Hi-C.

**B.** Mean H3.3 enrichment from asynchronous HIRA KO cells rescued with YFP (control, light green) and HIRA (green) quantified in A/B compartments per indicated domain sizes. Two-tailed Mann-Whitney U test adjusted for multiple testing by FDR (5% cut-off) was used to determine significance of differences between KO+YFP and HIRA rescue. Significance was noted as: * (p<=0.05), ** (p<=0.01), *** (p<=0.001) for all comparisons. The boxplot centre is the median, the bounds of the box are the first and third quartiles and the whiskers extend to 1.5x IQR. P-values are for KO+YFP vs HIRA rescue H3.3 asynchronous: A<=2Mb (p= 1.13e$^{-13}$), A>2Mb (p=0.94), B<=2Mb (p= 0.0003), B>2Mb (p= 0.001). Source data are provided as a Source data file.

**C.** Mean H3.3 enrichment from asynchronous WT (as reference) and HIRA KO cells rescued with YFP (control) and HIRA at blurred (n = 3274) and buried (n = 574) sites from 60-160kb in length.

**D.** Saddle plots from HIRA KO cells rescued with YFP (control) and HIRA (O/E contacts) and differential (log2 ratio of O/E contacts) of HIRA KO + HIRA/YFP rescue at 50kb resolution based on EV1 percentiles from H3.1- and H3.3-SNAP cells.

**E.** Scatterplots of mean EV1 value at buried sites (n = 439) from HIRA KO + YFP (control) and HIRA rescue coloured by their mean enrichment of H3.3 (left) or EdU at 2h in S (right) in HIRA KO + HIRA rescue.

H3.3 and EdU 2h in S enrichment was calculated at 10kb bins as z-score of $\log_2$ IP/input.

**Supplementary Table 1. Detection of TAD borders between cell line and condition**

| Detected in both cell lines | Detected in both conditions | Number of TAD borders | | | | Percentage of TAD borders | | | |
|---|---|---|---|---|---|---|---|---|---|
| | | H3.1 WT | H3.3 WT | H3.1 KO | H3.3 KO | H3.1 WT | H3.3 WT | H3.1 KO | H3.3 KO |
| **yes** | **yes** | 5413 | 5381 | 5385 | 5439 | 84,78 | 80,53 | 81,46 | 84,01 |
| **yes** | **no** | 411 | 443 | 489 | 435 | 6,44 | 6,63 | 7,4 | 6,72 |
| **no** | **yes** | 276 | 368 | 302 | 309 | 4,32 | 5,51 | 4,57 | 4,77 |
| **no** | **no** | 285 | 490 | 435 | 291 | 4,46 | 7,33 | 6,58 | 4,49 |
| **total** | | 6385 | 6682 | 6611 | 6474 | | | | |

**Supplementary Table 2. Overlap of ATAC-seq peaks common between cell lines and replicates between WT and HIRA KO**

| | Number of ATAC-seq peaks | | | Percentage of ATAC-seq peaks | |
|---|---|---|---|---|---|
| **Sample** | **all** | **shared (WT & KO)** | **unique (WT or KO)** | **shared (WT & KO)** | **unique (WT or KO)** |
| **H31_WT_r1** | 45630 | 41586 | 4044 | 91,14 | 8,86 |
| **H31_WT_r2** | 46777 | 44387 | 2390 | 94,89 | 5,11 |
| **H31_KO_r1** | 56885 | 42478 | 14407 | 74,67 | 25,33 |
| **H31_KO_r2** | 61588 | 47113 | 14475 | 76,5 | 23,5 |
| **H33_WT_r1** | 50357 | 44328 | 6029 | 88,03 | 11,97 |
| **H33_WT_r2** | 54418 | 48694 | 5724 | 89,48 | 10,52 |
| **H33_KO_r1** | 57287 | 49898 | 7389 | 87,1 | 12,9 |
| **H33_KO_r2** | 62913 | 53949 | 8964 | 85,75 | 14,25 |

**Supplementary Table 3. Materials**

| **Reagent** | **Source** | **Cat. No** |
|---|---|---|
| **Cell culture** | | |
| DMEM | ThermoFisher Scientific | 31966-021 |
| FBS | Eurobio | CVFSVF00-01 |
| PBS | ThermoFisher Scientific | 10010-056 |
| TrypLE | ThermoFisher Scientific | 12605-010 |
| **Transfection** | | |
| Opti-MEM I Reduced Serum Medium | Invistrogen | 31985062 |
| Lipofectamine 2000 | Invitrogen | 11668019 |
| Lipofectamine RNAiMAX | Thermo Fisher Scientific | 13778150 |
| YFP plasmid | Ray-Gallet et al., 2018[50] | |
| HIRA-YFP plasmid | Ray-Gallet et al., 2018[50] | |
| ON-TARGETplus Non-targeting Control siRNA | Dharmacon | D-001810-10 |
| ON-TARGETplus HIRA siRNA | Dharmacon | J-013610-06 |
| **Synchronisation** | | |
| Thymidine | Sigma-Aldrich | T1895 |
| 2'-deoxycytidine hydroxycloride | Sigma-Aldrich | D0776 |
| **IF** | | |
| Click-iT EdU Cell Proliferation kit | ThermoFisher | C10340 |

| | | | |
|---|---|---|---|
| DAPI (4',6-Diamidino-2-phenylindole) | Sigma | D9542 | |
| PFA (paraformaldehyde) | Euromedex | 15710 | |
| BSA (bovine serum albumin) | Sigma | A4503 | |
| Vectashield | Eurobio scientific | H-1000 | |
| **Antibodies** | | | |
| PCNA | DAKO | M879 | 1:1000 (IF) |
| H3K4me1 | abcam | ab8895 | 4ug (ChIP-seq) |
| H3K4me3 | Active Motif | 39915 | 4ug (ChIP-seq) |
| H3K9me3 | Active Motif | 39765 | 10ug (ChIP-seq) |
| H3K27ac | Active Motif | 39133 | 5ug (ChIP-seq) |
| H3K7me3 | Active Motif | 39155 | 5ug (ChIP-seq) |
| **ChIP-seq, SNAP-seq & EdU-seq** | | | |
| Complete EDTA free protease inhibitor cocktail 50x | Roche | 11873580001 | |
| SDS 2% | Euromedex | EU0660 | |
| Triton-X 10% | Euromedex | 2000-C | |
| Tween 20 | AMRESCO | 0777-1L | |
| NP40 | Euromedex | UN3500-A | |
| EGTA | Euromedex | 1310-B | |
| tRNA | Sigma | R7876-500 | |
| BSA (bovine serum albumin) | Euromedex | 04-100-812-C | |
| MNase | ThermoFisher Scientific | EN0181 | |
| RNase A, DNase free | Roche | 10109169001 | |
| Proteinase K | Roche | 03115879001 | |
| TSA | Sigma | T-8552 | |
| Agencourt AMPure XP beads | Beckman Coulter | A63880 | |
| Dynabeads Protein A | Invitrogen | 1002D | |
| Click-iT EdU Cell Proliferation kit | ThermoFisher | C10337 | |
| Biotin-PEG3-SS-azide | BroadPharm | BP-22955 | |
| Dynabeads M-280 Streptavidin | ThermoFisher Scientific | 11205D | |
| SNAP-Capture magnetic beads | New England Biolabs | S9145S | |
| D1000 high-sensitivity tape | Agilent | 5067-5584 | |
| D1000 high-sensitivity kit | Agilent | 5067-5585 | |
| Qubit dsDNA HS Assay Kit | Invitrogen | Q32854 | |
| TruSeq ChIP kit | Illumina | IP-202-1012 | |
| Low-binding tubes (Axygen) | VWR | MCT-150-LC | |
| Low-binding tips (Axygen) | VWR | TR-222-C-L-R | |
| **Hi-C** | | | |
| 37% formaldehyde (MeOH-stabilised) | Sigma | F8775 | |
| Arima Hi-C+ kit | Arima Genomics | N/A | |
| KAPA HyperPrep kit | Roche | 7962312001 | |
| KAPA Library Quantification Sample kit | Roche | 7960166001 | |
| TruSeq DNA UD indexes (24UDD, 96 Samples) | Illumina | 20020590 | |
| **Other kits** | | | |
| ATAC-seq kit | Active Motif | 53150 | |

| | | | |
|---|---|---|---|
| RNeasy Plus kit | QIAGEN | 74134 | |
| DNase | QIAGEN | 79254 | |
| ERCC ExFold RNA Spike-In Mix | Invitrogen | 4456739 | |
| TruSeq Stranded Total RNA kit | Illumina | 20020596 | |
| **Software** | | **version** | **used for** |
| bowtie2 | Langmead and Salzberg, 2012[82] | 2.3.4.2 | alignment (ChIP-seq, ATAC-seq) |
| hisat2 | Kim et al., 2019[83] | 2.1.0 | alignment (RNA-seq) |
| SAMtools | Danecek et al., 2021[84] | 1.9 | bam file processing |
| ATACseqQC | Ou et al., 2018[85] | 1.28.0 | ATAC-seq QC |
| BEDtools | Quinlan and Hall, 2010[86] | 2.27.1 | bed file processing |
| HiC-Pro | Servant et al., 2015[93] | 3.1.0 | Hi-C matrix generation |
| multiQC | Ewels et al., 2016[94] | 1.11 | Hi-C matrix QC |
| HiCExplorer | Ramírez et al., 2018[95]; Wolff et al., 2020[96], 2018[97] | 3.7.2 | Hi-C filetype conversion |
| HiGlass | Kerpedjiev et al., 2018[98] | 0.8.0 | interactive matrix visualization |
| HMMRATAC | Tarbell and Liu, 2019[92] | 1.2.10 | ATAC-seq peak calling |
| python | | 3.10.12 | analysis |
| pandas | McKinney, 2010[87] | 1.5.3 | table operations |
| numpy | Harris et al., 2020[88] | 1.23.5 | matrix operations |
| scipy | Virtanen et al., 2020[89] | 1.11.2 | correlation, statistical analysis |
| matplotlib | Hunter, 2007[90] | 3.6.2 | plotting |
| seaborn | Waskom, 2021[91] | 0.12.2 | plotting |
| bioframe | Open2C et al., 2022[103] | 0.4.1 | chromosome arm annotation |
| cooler | Abdennur and Mirny, 2020[100] | 0.9.1 | Hi-C matrix normalisation |
| cooltools | Open 2C et al., 2022[103] | 0.6.1 | Hi-C matrix analysis and plotting |
| HiCrep | Lin et al., 2021[101]; Yang et al., 2017[102] | 0.2.6 | Hi-C matrix similarity |
| **Publicly available data** | | | |
| Human reference genome | GRCh38 | | |
| Blacklisted regions (ensembl) | GRCh38-blacklist.v2 + manual curation | | |
| H3.1- and H3.3- SNAP-seq at G1/S + EdU 2h | Gatto et al., 2022[19] | E-MTAB-10619 | |
| H3.3 site locations & H3.3+ OK-seq IZs | Gatto et al., 2022[19] | E-MTAB-10619 | |
