# Supplementary material for: HIRA defines early replication initiation zones independently of their genome compartment
Source: Nat Commun. 2025 Nov 6;16:9715. doi: 10.1038/s41467-025-65130-2 (PMC12592364; doi:10.1038/s41467-025-65130-2)
Supplement: Supplementary file 2 — Reporting Summary [file 41467_2025_65130_MOESM2_ESM.pdf]

nature portfolio

Corresponding author(s): Almouzni Genevève

Last updated by author(s): Jun 9, 2025

Reporting Summary

Nature Portfolio wishes to improve the reproducibility of the work that we publish. This form provides structure for consistency and transparency in reporting. For further information on Nature Portfolio policies, see our Editorial Policies and the Editorial Policy Checklist.

Statistics

For all statistical analyses, confirm that the following items are present in the figure legend, table legend, main text, or Methods section.

n/a	Confirmed	
☐	☒	The exact sample size (*n*) for each experimental group/condition, given as a discrete number and unit of measurement
☐	☒	A statement on whether measurements were taken from distinct samples or whether the same sample was measured repeatedly
☐	☒	The statistical test(s) used AND whether they are one- or two-sided<br>*Only common tests should be described solely by name; describe more complex techniques in the Methods section.*
☒	☐	A description of all covariates tested
☐	☒	A description of any assumptions or corrections, such as tests of normality and adjustment for multiple comparisons
☐	☒	A full description of the statistical parameters including central tendency (e.g. means) or other basic estimates (e.g. regression coefficient) AND variation (e.g. standard deviation) or associated estimates of uncertainty (e.g. confidence intervals)
☐	☒	For null hypothesis testing, the test statistic (e.g. *F*, *t*, *r*) with confidence intervals, effect sizes, degrees of freedom and *P* value noted<br>*Give P values as exact values whenever suitable.*
☒	☐	For Bayesian analysis, information on the choice of priors and Markov chain Monte Carlo settings
☒	☐	For hierarchical and complex designs, identification of the appropriate level for tests and full reporting of outcomes
☒	☐	Estimates of effect sizes (e.g. Cohen's *d*, Pearson's *r*), indicating how they were calculated

Our web collection on statistics for biologists contains articles on many of the points above.

Software and code

Policy information about availability of computer code

Data collection	No software was used in this study
Data analysis	ChIP-seq and ATAC-seq raw reads (in FASTQ format) were mapped to the soft-masked human reference genome (GRCh38, release 109, ensembl) using bowtie2 v2.3.4.2 (Langmead and Salzberg, 2012) with --very-sensitive parameters. RNA-seq data was aligned with hisat2 v2.1.0 (Kim et al., 2019), run in paired-end mode with default parameters. We used SAMtools v1.9 (Danecek et al., 2021) to sort, flag duplicates and index bam files for all samples. We used samtools view (-f 2 -F 3840 parameters to keep reads mapped in pairs and exclude QC fails, non-primary alignments and duplicates) to compute coverage over the genome as a BED file (chromosome, start, end, MAPQ). Quality control of ATAC-seq data was additionally performed on bam files by ATACseqQC v1.28.0 (Ou et al., 2018). We used BEDtools v2.27.1 (Quinlan and Hall, 2010) to calculate number of fragments in consecutive 100bp or 1kb bins. ATAC-seq peaks were called from each replicate and condition using HMMRATAC (Tarbell and Liu, 2019) with default parameters except --window 2500000.<br>We used HiC-Pro v3.1.0 (Servant et al., 2015) with default parameters (except MIN_MAPQ=2) to generate raw count Hi-C matrices at 1Mb, 100kb, 50kb, 25kb, 10kb and 5kb resolution from raw FASTQ files. First, we used MultiQC v1.11 (Ewels et al., 2016) to perform quality control and extract the number of short-range (<=20kb), long-range (>20kb) cis and trans interactions for each sample. HiCExplorer v3.7.2 (Ramírez et al., 2018; Wolff et al., 2020, 2018) was then used to convert matrices to cool format and generate a single mcool file per sample, containing all resolutions listed above. Matrices were visualized interactively with HiGlass v0.8.0 (Kerpedjiev et al., 2018) at 1Mb and 100kb resolutions to be manually inspected for large inter- and intra-chromosomal aberrations (translocations, inversions, duplications, etc.), and the subsequently generated list of regions was merged with the set of blacklisted regions of the human genome (ensembl). This custom set of blacklisted regions was used to mask matrices prior to normalization by iterative correction (ICE) (Imakaev et al., 2012) with a single iteration using cooler v0.9.3 (Abdennur and Mirny, 2020). Matrix similarity was computed per chromosome at 1Mb, 100kb, 50kb and 10kb resolution with HiCRep v0.2.6 (Lin et al., 2021; Yang et al., 2017) before and after masking without substantial changes. Expected interactions per chromosome arm (coordinates downloaded with bioframe) were calculated using cooltools v0.6.1 (Open 2C et al., 2022). Compartment

analysis of Hi-C matrices was performed by eigenvector (EV) decomposition (Lieberman-Aiden et al., 2009) at 50kb resolution with cooltools using GC content track to orient the sign of the first eigenvector (EV1). TAD borders were identified based on insulation score (Crane et al., 2015), computed from 10kb binned matrices with a window size of 100kb using cooltools.
Analysis of the data was carried out by custom Python scripts using pandas v1.5.3 (McKinney, 2010), NumPy v1.23.5 (Harris et al., 2020) and scipy v1.11.2 (Virtanen et al., 2020). Visualization was performed using matplotlib v3.6.2 (Hunter, 2007) and seaborn v0.12.2 (Waskom, 2021).
Statistical analysis was performed in python using scipy (Virtanen et al., 2020).
We used Fiji (v1.54i) software for microscopy image visualization and analysis.
Additional information is available in the Methods section.

For manuscripts utilizing custom algorithms or software that are central to the research but not yet described in published literature, software must be made available to editors and reviewers. We strongly encourage code deposition in a community repository (e.g. GitHub). See the Nature Portfolio guidelines for submitting code & software for further information.

Data

Policy information about availability of data

All manuscripts must include a data availability statement. This statement should provide the following information, where applicable:

- Accession codes, unique identifiers, or web links for publicly available datasets
- A description of any restrictions on data availability
- For clinical datasets or third party data, please ensure that the statement adheres to our policy

Data from sequencing experiments performed in this study have been deposited on ArrayExpress with the following accession numbers: E-MTAB-14416 (H3 PTM ChIP-seq), E-MTAB-14415 (ATAC-seq), E-MTAB-14417 (RNA-seq), E-MTAB-14433 (Hi-C), E-MTAB-14419 (SNAP-seq and EdU-seq upon HIRA rescue) and E-MTAB-14431 (Hi-C upon HIRA rescue). This paper does not report original code. Source data is provided. Any additional information is available from the lead contact upon reasonable request.

Research involving human participants, their data, or biological material

Policy information about studies with human participants or human data. See also policy information about sex, gender (identity/presentation), and sexual orientation and race, ethnicity and racism.

Reporting on sex and gender	*Use the terms sex (biological attribute) and gender (shaped by social and cultural circumstances) carefully in order to avoid confusing both terms. Indicate if findings apply to only one sex or gender; describe whether sex and gender were considered in study design; whether sex and/or gender was determined based on self-reporting or assigned and methods used. Provide in the source data disaggregated sex and gender data, where this information has been collected, and if consent has been obtained for sharing of individual-level data; provide overall numbers in this Reporting Summary. Please state if this information has not been collected. Report sex- and gender-based analyses where performed, justify reasons for lack of sex- and gender-based analysis.*
Reporting on race, ethnicity, or other socially relevant groupings	*Please specify the socially constructed or socially relevant categorization variable(s) used in your manuscript and explain why they were used. Please note that such variables should not be used as proxies for other socially constructed/relevant variables (for example, race or ethnicity should not be used as a proxy for socioeconomic status). Provide clear definitions of the relevant terms used, how they were provided (by the participants/respondents, the researchers, or third parties), and the method(s) used to classify people into the different categories (e.g. self-report, census or administrative data, social media data, etc.) Please provide details about how you controlled for confounding variables in your analyses.*
Population characteristics	*Describe the covariate-relevant population characteristics of the human research participants (e.g. age, genotypic information, past and current diagnosis and treatment categories). If you filled out the behavioural & social sciences study design questions and have nothing to add here, write "See above."*
Recruitment	*Describe how participants were recruited. Outline any potential self-selection bias or other biases that may be present and how these are likely to impact results.*
Ethics oversight	*Identify the organization(s) that approved the study protocol.*

Note that full information on the approval of the study protocol must also be provided in the manuscript.

Field-specific reporting

Please select the one below that is the best fit for your research. If you are not sure, read the appropriate sections before making your selection.

☒ Life sciences ☐ Behavioural & social sciences ☐ Ecological, evolutionary & environmental sciences

For a reference copy of the document with all sections, see nature.com/documents/nr-reporting-summary-flat.pdf

Life sciences study design

All studies must disclose on these points even when the disclosure is negative.

Sample size | All sequencing experiments were performed at least in two biological replicates. For microscopy, sample size was defined according to the

Sample size	maximum amount of cells observed per microscopic slide.
Data exclusions	No data was excluded in this study.
Replication	All experiments have been replicated. The number of replicates is indicated in the text.
Randomization	No randomization was needed for this study
Blinding	For microscopy experiments, blinding was done during analysis (counting number of early-replicating cells following S-phase release). Blinding could not be done for counting number of transfected cells because YFP and HIRA-YFP have different staining patterns. For sequencing experiments, blinding could not be performed.

Reporting for specific materials, systems and methods

We require information from authors about some types of materials, experimental systems and methods used in many studies. Here, indicate whether each material, system or method listed is relevant to your study. If you are not sure if a list item applies to your research, read the appropriate section before selecting a response.

Materials & experimental systems

n/a	Involved in the study
☐	☒ Antibodies
☐	☒ Eukaryotic cell lines
☒	☐ Palaeontology and archaeology
☒	☐ Animals and other organisms
☒	☐ Clinical data
☒	☐ Dual use research of concern
☒	☐ Plants

Methods

n/a	Involved in the study
☐	☒ ChIP-seq
☒	☐ Flow cytometry
☒	☐ MRI-based neuroimaging

Antibodies

Antibodies used	PCNA (DAKO, M879), H3K4me1 (Abcam, ab8895), H3K4me3 (Active Motif, 39915), H3K9me3 (Active Motif, 39765), H3K27ac (Active Motif, 39133), H3K7me3 (Active Motif, 39155). Information on dilution/amount used is reported in the Materials table.
Validation	These antibodies have been already characterized in previous publications from our group or others.

Eukaryotic cell lines

Policy information about cell lines and Sex and Gender in Research

Cell line source(s)	HeLa cells stably expressing H3.1-SNAP-HA or H3.3-SNAP-HA that were either wild-type (WT) or HIRA knock out (KO) (CRISPR/Cas9-mediated), established in (Ray-Gallet et al., 2018) and U2OS cells (ATCC HTB-96) were used in this study.
Authentication	None of the cells used were authenticated in this study
Mycoplasma contamination	Cell lines used in this study were tested negative for mycoplasma contamination.
Commonly misidentified lines (See ICLAC register)	This study did not involve misidentified cell lines.

Plants

Seed stocks	*Report on the source of all seed stocks or other plant material used. If applicable, state the seed stock centre and catalogue number. If plant specimens were collected from the field, describe the collection location, date and sampling procedures.*
Novel plant genotypes	*Describe the methods by which all novel plant genotypes were produced. This includes those generated by transgenic approaches, gene editing, chemical/radiation-based mutagenesis and hybridization. For transgenic lines, describe the transformation method, the number of independent lines analyzed and the generation upon which experiments were performed. For gene-edited lines, describe the editor used, the endogenous sequence targeted for editing, the targeting guide RNA sequence (if applicable) and how the editor was applied.*
Authentication	*Describe any authentication procedures for each seed stock used or novel genotype generated. Describe any experiments used to assess the effect of a mutation and, where applicable, how potential secondary effects (e.g. second site T-DNA insertions, mosiacism, off-target gene editing) were examined.*

ChIP-seq

Data deposition

☒ Confirm that both raw and final processed data have been deposited in a public database such as GEO.

☐ Confirm that you have deposited or provided access to graph files (e.g. BED files) for the called peaks.

Data access links<br>*May remain private before publication.*	Data from sequencing experiments performed in this study have been deposited on ArrayExpress with the following accession numbers: E-MTAB-14416 (H3 PTM ChIP-seq), E-MTAB-14415 (ATAC-seq), E-MTAB-14417 (RNA-seq), E-MTAB-14433 (Hi-C), E-MTAB-14419 (SNAP-seq and EdU-seq upon HIRA rescue) and E-MTAB-14431 (Hi-C upon HIRA rescue).

Files in database submission

E-MTAB-14415,ATAC-seq
KO_H31SNAP_r1,ERS20948786.R1.fastq,ERS20948786.R2.fastq,ATAC-seq
KO_H31SNAP_r2,ERS20948787.R1.fastq,ERS20948787.R2.fastq,ATAC-seq
KO_H33SNAP_r1,ERS20948788.R1.fastq,ERS20948788.R2.fastq,ATAC-seq
KO_H33SNAP_r2,ERS20948789.R1.fastq,ERS20948789.R2.fastq,ATAC-seq
WT_H31SNAP_r1,ERS20948790.R1.fastq,ERS20948790.R2.fastq,ATAC-seq
WT_H31SNAP_r1,ERS20948790.R2.fastq,ERS20948791.R1.fastq,ATAC-seq
WT_H31SNAP_r2,ERS20948791.R2.fastq,ERS20948792.R1.fastq,ATAC-seq
WT_H33SNAP_r1,ERS20948792.R1.fastq,ERS20948792.R2.fastq,ATAC-seq
WT_H33SNAP_r2,ERS20948793.R1.fastq,ERS20948793.R2.fastq,ATAC-seq
E-MTAB-14416,H3 PTM ChIP-Seq
KO_H31SNAP_input,ERS20948818.R1.fastq,ERS20948818.R2.fastq,H3 PTM ChIP-Seq
WT_H31SNAP_input,ERS20948819.R1.fastq,ERS20948819.R2.fastq,H3 PTM ChIP-Seq
H3K27me3_WT_H33SNAP_input,ERS20948803.R1.fastq,ERS20948803.R2.fastq,H3 PTM ChIP-Seq
H3K4me3_KO_H33SNAP_input,ERS20948810.R1.fastq,ERS20948810.R2.fastq,H3 PTM ChIP-Seq
all_others_KO_H33SNAP_input,ERS20948794.R1.fastq,ERS20948794.R2.fastq,H3 PTM ChIP-Seq
all_others_WT_H33SNAP_input,ERS20948795.R1.fastq,ERS20948795.R2.fastq,H3 PTM ChIP-Seq
H3K27ac_KO_H31SNAP_IP,ERS20948796.R1.fastq,ERS20948796.R2.fastq,H3 PTM ChIP-Seq
H3K27ac_KO_H33SNAP_IP,ERS20948797.R1.fastq,ERS20948797.R2.fastq,H3 PTM ChIP-Seq
H3K27ac_WT_H31SNAP_IP,ERS20948798.R1.fastq,ERS20948798.R2.fastq,H3 PTM ChIP-Seq
H3K27ac_WT_H33SNAP_IP,ERS20948799.R1.fastq,ERS20948799.R2.fastq,H3 PTM ChIP-Seq
H3K27me3_KO_H31SNAP_IP,ERS20948800.R1.fastq,ERS20948800.R2.fastq,H3 PTM ChIP-Seq
H3K27me3_KO_H33SNAP_IP,ERS20948801.R1.fastq,ERS20948801.R2.fastq,H3 PTM ChIP-Seq
H3K27me3_WT_H31SNAP_IP,ERS20948802.R1.fastq,ERS20948802.R2.fastq,H3 PTM ChIP-Seq
H3K27me3_WT_H33SNAP_IP,ERS20948804.R1.fastq,ERS20948804.R2.fastq,H3 PTM ChIP-Seq
H3K4me1_KO_H31SNAP_IP,ERS20948805.R1.fastq,ERS20948805.R2.fastq,H3 PTM ChIP-Seq
H3K4me1_KO_H33SNAP_IP,ERS20948806.R1.fastq,ERS20948806.R2.fastq,H3 PTM ChIP-Seq
H3K4me1_WT_H31SNAP_IP,ERS20948807.R1.fastq,ERS20948807.R2.fastq,H3 PTM ChIP-Seq
H3K4me1_WT_H33SNAP_IP,ERS20948808.R1.fastq,ERS20948808.R2.fastq,H3 PTM ChIP-Seq
H3K4me3_KO_H31SNAP_IP,ERS20948809.R1.fastq,ERS20948809.R2.fastq,H3 PTM ChIP-Seq
H3K4me3_KO_H33SNAP_IP,ERS20948811.R1.fastq,ERS20948811.R2.fastq,H3 PTM ChIP-Seq
H3K4me3_WT_H31SNAP_IP,ERS20948812.R1.fastq,ERS20948812.R2.fastq,H3 PTM ChIP-Seq
H3K4me3_WT_H33SNAP_IP,ERS20948813.R1.fastq,ERS20948813.R2.fastq,H3 PTM ChIP-Seq
H3K9me3_KO_H31SNAP_IP,ERS20948814.R1.fastq,ERS20948814.R2.fastq,H3 PTM ChIP-Seq
H3K9me3_KO_H33SNAP_IP,ERS20948815.R1.fastq,ERS20948815.R2.fastq,H3 PTM ChIP-Seq
H3K9me3_WT_H31SNAP_IP,ERS20948816.R1.fastq,ERS20948816.R2.fastq,H3 PTM ChIP-Seq
H3K9me3_WT_H33SNAP_IP,ERS20948817.R1.fastq,ERS20948817.R2.fastq,H3 PTM ChIP-Seq
E-MTAB-14417,RNA-seq
KO_H31SNAP_r1,ERS20948820.R1.fastq,ERS20948820.R2.fastq,RNA-Seq
KO_H31SNAP_r2,ERS20948821.R1.fastq,ERS20948821.R2.fastq,RNA-Seq
KO_H33SNAP_r1,ERS20948822.R1.fastq,ERS20948822.R2.fastq,RNA-Seq
KO_H33SNAP_r2,ERS20948823.R1.fastq,ERS20948823.R2.fastq,RNA-Seq
WT_H31SNAP_r1,ERS20948824.R1.fastq,ERS20948824.R2.fastq,RNA-Seq
WT_H31SNAP_r2,ERS20948825.R1.fastq,ERS20948825.R2.fastq,RNA-Seq
WT_H33SNAP_r1,ERS20948826.R1.fastq,ERS20948826.R2.fastq,RNA-Seq
WT_H33SNAP_r2,ERS20948827.R1.fastq,ERS20948827.R2.fastq,RNA-Seq
E-MTAB-14419,EdU-seq and SNAP-seq HIRA rescue
EdU_H31SNAP_HIRA_2h_input_r1,ERS20948828.R1.fastq,ERS20948828.R2.fastq,EdU-Seq
EdU_H31SNAP_HIRA_2h_input_r2,ERS20948829.R1.fastq,ERS20948829.R2.fastq,EdU-Seq
EdU_H31SNAP_HIRA_2h_IP_r1,ERS20948830.R1.fastq,ERS20948830.R2.fastq,EdU-Seq
EdU_H31SNAP_HIRA_2h_IP_r2,ERS20948831.R1.fastq,ERS20948831.R2.fastq,EdU-Seq
EdU_H31SNAP_YFP_2h_input_r1,ERS20948832.R1.fastq,ERS20948832.R2.fastq,EdU-Seq
EdU_H31SNAP_YFP_2h_input_r2,ERS20948833.R1.fastq,ERS20948833.R2.fastq,EdU-Seq
EdU_H31SNAP_YFP_2h_IP_r1,ERS20948834.R1.fastq,ERS20948834.R2.fastq,EdU-Seq
EdU_H31SNAP_YFP_2h_IP_r2,ERS20948835.R1.fastq,ERS20948835.R2.fastq,EdU-Seq
EdU_H33SNAP_HIRA_2h_input_r2,ERS20948836.R1.fastq,ERS20948836.R2.fastq,EdU-Seq
EdU_H33SNAP_HIRA_2h_IP_r1,ERS20948837.R1.fastq,ERS20948837.R2.fastq,EdU-Seq
EdU_H33SNAP_HIRA_2h_IP_r2,ERS20948838.R1.fastq,ERS20948838.R2.fastq,EdU-Seq
EdU_H33SNAP_YFP_2h_input_r1,ERS20948839.R1.fastq,ERS20948839.R2.fastq,EdU-Seq
EdU_H33SNAP_YFP_2h_input_r2,ERS20948840.R1.fastq,ERS20948840.R2.fastq,EdU-Seq
EdU_H33SNAP_YFP_2h_IP_r1,ERS20948841.R1.fastq,ERS20948841.R2.fastq,EdU-Seq
EdU_H33SNAP_YFP_2h_IP_r2,ERS20948842.R1.fastq,ERS20948842.R2.fastq,EdU-Seq

H31SNAP_HIRA_0h_input_r1,ERS20948843.R1.fastq,ERS20948843.R2.fastq,SNAP-Seq
H31SNAP_HIRA_0h_input_r2,ERS20948844.R1.fastq,ERS20948844.R2.fastq,SNAP-Seq
H31SNAP_HIRA_0h_IP_r1,ERS20948845.R1.fastq,ERS20948845.R2.fastq,SNAP-Seq
H31SNAP_HIRA_0h_IP_r2,ERS20948846.R1.fastq,ERS20948846.R2.fastq,SNAP-Seq
H31SNAP_YFP_0h_input_r1,ERS20948847.R1.fastq,ERS20948847.R2.fastq,SNAP-Seq
H31SNAP_YFP_0h_input_r2,ERS20948848.R1.fastq,ERS20948848.R2.fastq,SNAP-Seq
H31SNAP_YFP_0h_IP_r1,ERS20948849.R1.fastq,ERS20948849.R2.fastq,SNAP-Seq
H31SNAP_YFP_0h_IP_r2,ERS20948850.R1.fastq,ERS20948850.R2.fastq,SNAP-Seq
H33SNAP_HIRA_0h_input_r1,ERS20948851.R1.fastq,ERS20948851.R2.fastq,SNAP-Seq
H33SNAP_HIRA_0h_input_r2,ERS20948852.R1.fastq,ERS20948852.R2.fastq,SNAP-Seq
H33SNAP_HIRA_0h_IP_r1,ERS20948853.R1.fastq,ERS20948853.R2.fastq,SNAP-Seq
H33SNAP_HIRA_0h_IP_r2,ERS20948854.R1.fastq,ERS20948854.R2.fastq,SNAP-Seq
H33SNAP_HIRA_asynch_input_r1,ERS20948855.R1.fastq,ERS20948855.R2.fastq,SNAP-Seq
H33SNAP_HIRA_asynch_input_r2,ERS20948856.R1.fastq,ERS20948856.R2.fastq,SNAP-Seq
H33SNAP_HIRA_asynch_IP_r1,ERS20948857.R1.fastq,ERS20948857.R2.fastq,SNAP-Seq
H33SNAP_HIRA_asynch_IP_r2,ERS20948858.R1.fastq,ERS20948858.R2.fastq,SNAP-Seq
H33SNAP_YFP_0h_input_r1,ERS20948859.R1.fastq,ERS20948859.R2.fastq,SNAP-Seq
H33SNAP_YFP_0h_input_r2,ERS20948860.R1.fastq,ERS20948860.R2.fastq,SNAP-Seq
H33SNAP_YFP_0h_IP_r1,ERS20948861.R1.fastq,ERS20948861.R2.fastq,SNAP-Seq
H33SNAP_YFP_0h_IP_r2,ERS20948862.R1.fastq,ERS20948862.R2.fastq,SNAP-Seq
H33SNAP_YFP_asynch_input_r1,ERS20948863.R1.fastq,ERS20948863.R2.fastq,SNAP-Seq
H33SNAP_YFP_asynch_input_r2,ERS20948864.R1.fastq,ERS20948864.R2.fastq,SNAP-Seq
H33SNAP_YFP_asynch_IP_r1,ERS20948865.R1.fastq,ERS20948865.R2.fastq,SNAP-Seq
H33SNAP_YFP_asynch_IP_r2,ERS20948866.R1.fastq,ERS20948866.R2.fastq,SNAP-Seq
E-MTAB-14433,Hi-C HIRA WT vs KO
HiC_KO_H31SNAP,ERS20953405.R1.fastq,ERS20953405.R2.fastq,Hi-C
HiC_KO_H33SNAP,ERS20953406.R1.fastq,ERS20953406.R2.fastq,Hi-C
HiC_WT_H31SNAP,ERS20953407.R1.fastq,ERS20953407.R2.fastq,Hi-C
HiC_WT_H33SNAP,ERS20953408.R1.fastq,ERS20953408.R2.fastq,Hi-C
E-MTAB-14431,Hi-C HIRA rescue
HiC_KO_HIRA_H31SNAP,ERS20953401.R1.fastq,ERS20953401.R2.fastq,Hi-C
HiC_KO_HIRA_H33SNAP,ERS20953402.R1.fastq,ERS20953402.R2.fastq,Hi-C
HiC_KO_YFP_H31SNAP,ERS20953403.R1.fastq,ERS20953403.R2.fastq,Hi-C
HiC_KO_YFP_H33SNAP,ERS20953404.R1.fastq,ERS20953404.R2.fastq,Hi-C

Genome browser session
(e.g. UCSC)

Provide a link to an anonymized genome browser session for "Initial submission" and "Revised version" documents only, to enable peer review. Write "no longer applicable" for "Final submission" documents.

Methodology

Replicates

All sequencing experiments were performed at least in 2 biological relpicates (labelled H31SNAP and H33SNAP). Technical replicates were also performed for ATAC-seq, EdU-seq, SNAP-seq and RNA-seq (labelled r1/r2).

Sequencing depth

We performed PE100 sequencing on Illumina NovaSeq 6000 to at least 80M reads/sample except for Hi-C (350M reads/sample).

Antibodies

H3K4me1 (Abcam, ab8895), H3K4me3 (Active Motif, 39915), H3K9me3 (Active Motif, 39765), H3K27ac (Active Motif, 39133), H3K7me3 (Active Motif, 39155). Information on dilution/amount used is reported in the Materials table.

Peak calling parameters

We did not perform peak calling from the ChIP-seq data.

Data quality

We performed quality control of fastq files using MultiQC v1.11 (Ewels et al., 2016) for Hi-C experiments and fastQC (Andrews, 2010) for all other sequencing experiments. We used HiCRep v0.2.6 (Lin et al., 2021; Yang et al., 2017) to compare matrices between biological replicates and pearson correlation to compare ChIP-seq tracks between replicates genome-wide.

Software

Read mapping and preprocessing: bowtie2 v2.3.4.2 (Langmead, 2012), SAMtools v1.9 (Danecek, 2021)
Read quantification and plotting: python 3.9, pandas 1.5.3 (McKinney, 2010), numpy 1.23.5 (Harris et al., 2020), scipy 1.11.2 (Virtanen et al., 2020), matplotlib 3.6.2 (Hunter, 2007)
